# Cell cycle checkpoint revolution: targeted therapies in the fight against malignant tumors

**DOI:** 10.3389/fphar.2024.1459057

**Published:** 2024-10-11

**Authors:** Guangming Song, Jue Liu, Xing Tang, Jie Zhong, Yuhuan Zeng, Xiaodi Zhang, Jianbin Zhou, Jie Zhou, Lu Cao, Qunfeng Zhang, Yukun Li

**Affiliations:** ^1^ Department of Obstetrics and Gynecology, The Second Affiliated Hospital, Hengyang Medical School, University of South China, Hengyang, Hunan, China; ^2^ Department of Assisted Reproductive Centre, The affiliated Zhuzhou hospital Xiangya medical college, Central South University, Zhuzhou, Hunan, China

**Keywords:** malignant tumor, cell cycle, cell cycle checkpoint, cyclin, cyclin-dependent kinase, chemotherapeutic drug

## Abstract

Malignant tumors are among the most important causes of death worldwide. The pathogenesis of a malignant tumor is complex and has not been fully elucidated. Studies have shown that such pathogenesis is related to abnormal cell cycle progression. The expression levels of cyclins, cyclin-dependent kinases (CDKs), and CDK inhibitors as well as functions of the cell cycle checkpoints determine whether the cell cycle progression is smooth. Cell-cycle-targeting drugs have the advantages of high specificity, low toxicity, low side effects, and low drug resistance. Identifying drugs that target the cell cycle and applying them in clinical treatments are expected to promote chemotherapeutic developments against malignant tumors. This article aims to review drugs targeted against the cell cycle and their action mechanisms.

## 1 Introduction

The cell cycle refers to a series of events within a cell that cause it to divide into two new daughter cells. The typical cell cycle is divided into four phases, namely, G1, S, G2, and M phases. The function of the G1 phase is to prepare for the S phase and synthesize large amounts of RNA and proteins. The S phase mainly involves DNA replication. Small amounts of RNA and proteins are synthesized in the G2 phase. Finally, the cells undergo karyokinesis and cytokinesis in the M phase. In the process of cell life, various factors (environmental factors, self-factors, etc.) can easily affect the integrity of the genetic material of the cell (Barnum and O’Connell, 2014; Li et al., 2022). Cells have developed a series of regulatory mechanisms to ensure continuous cell division and accurate replication of the cellular genetic material known as the cell cycle regulatory system.

The cell cycle consists of three important nodes that we refer to as the cell cycle checkpoints. The first is the G1/S checkpoint between the G1 and S phases and is also called as the restriction point. The second is the G2/M checkpoint located between the G2 and M phases and is also called as the DNA damage checkpoint. The third is the mitotic spindle assembly checkpoint (SAC) (Khan and Wang, 2022). The cell cycle can function properly only through these checkpoints (Poon, 2016), and the three most critical types of proteins involved in regulating the cell cycle are the cyclins, cyclin-dependent kinases (CDKs), and CDK inhibitor proteins (Khan and Wang, 2022). Cyclins bind to the CDKs to form complexes that drive the cell cycle (Gao et al., 2020). Different complexes can act on different phases of the cell cycle. For example, the cyclinD–CDK4/6 and cyclinE–CDK2 complexes act on the G1 phase; the cyclinA–CDK2 complex acts on the S phase; the cyclinB–CDK1 complex acts on the G2 and M phases (Pan et al., 2023).

Malignant tumors are among the most important causes of death worldwide, and their incidence is increasing annually. In the 21st century, cancer is expected to become the leading cause of death in every country and imposes an enormous burden (Lin et al., 2021). The treatments for malignant tumors include surgery, radiotherapy, and chemotherapy; however, patients can relapse easily after treatment, and the mortality rate is high, which is a major problem for not only clinicians but also researchers. Studies have shown that the pathogenesis of a malignant tumor is related to abnormal cell cycle progression (Liu et al., 2022), and the search for targeted drugs acting on the cell cycle is expected to provide new avenues for the treatment of malignant tumors.

Herein, our main purpose is to explore the relationships between malignant tumors and cell cycle regulation as well as between the cell cycle and targeted drugs (Table 1); we also review these relationships to provide references and evidence for subsequent research on malignant tumors.

**TABLE 1:** Cell-cycle-targeting drugs and tumor types treated.

Classification of the drug	Action period	Name of drug	Ki/IC50	Chemical structure	Tumor type	Reference
ATM inhibitors	S phase	AZD0156	IC50 = 0.58 nM	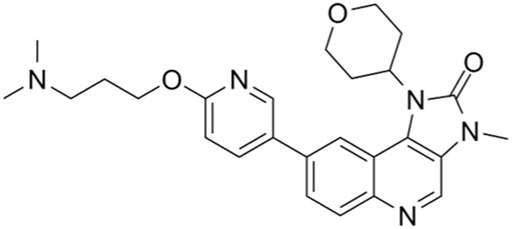	Advanced solid tumors	Imidazo 4,5-c quinolin-2-one compounds and their use in treating cancer (2019)
		AZD1390	IC50 = 0.78 nM	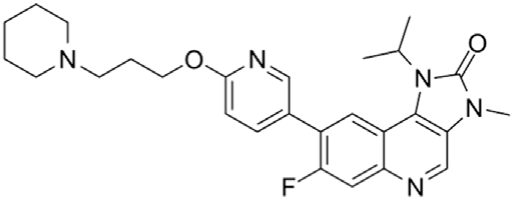	Brain tumors	Durant et al. (2018)
CDK2 inhibitors	S phase	Indisulam (E7070)	Wide range of IC50 values	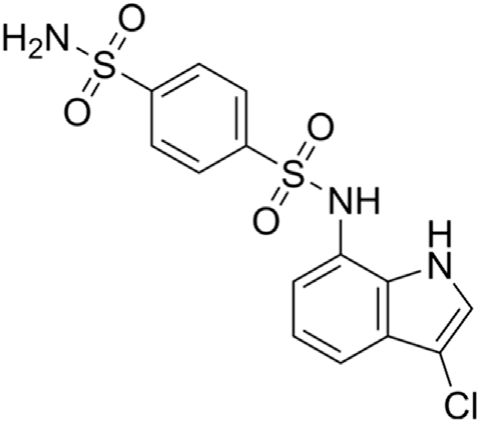	Lung cancer	Ozawa et al. (2001)
CDK4/6 inhibitors	G1 phase	Palbociclib	IC50 = 11 nM or 16 nM	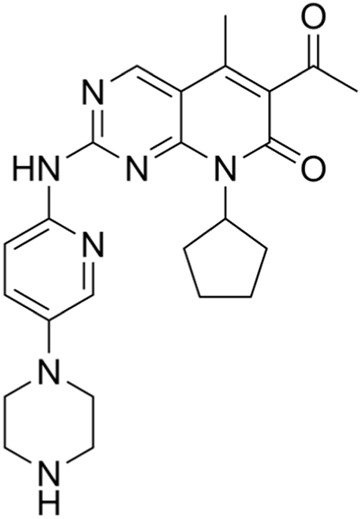	Breast cancer, lymphoma, etc.	Fry et al. (2004)
		Ribociclib	IC50 = 10 nM or 39 nM	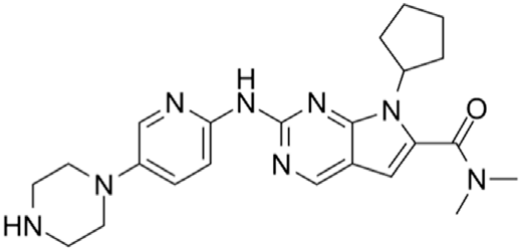	Breast cancer, liposarcoma, head and neck squamous cell carcinomas, melanoma, neuroblastoma, etc.	VanArsdale et al. (2015)
		Abemaciclib	IC50 = 2 nM or 10 nM	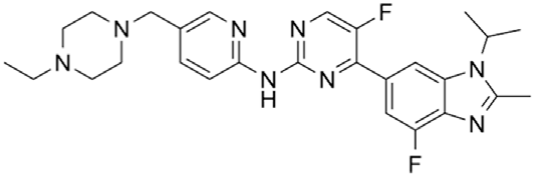	Breast cancer	Gelbert et al. (2014)
ATR inhibitors	S and G2 phases	M6620 (VX-970)	Ki = 0.2 nM	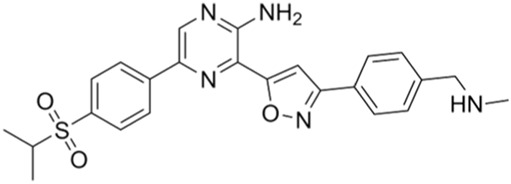	Brain metastases, solid tumors, esophageal cancer, ovarian cancer, primary peritoneal cancer, fallopian tube cancer, metastatic urothelial cancer, prostate cancer, metastatic gastric cancer, etc.	Fokas et al. (2012)
		Ceralasertib (AZD6738)	IC50 = 1 nM	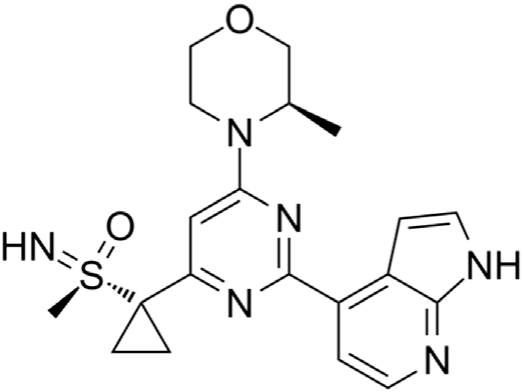	CLL, PLL, B-cell lymphoma, CML, MDS, NHL, head and neck squamous cell carcinoma, NSCLC, gastric cancer, TNBC, renal cell carcinoma, urothelial carcinoma, pancreatic cancer, SCLC, prostate cancer, melanoma advanced solid tumor, metastatic tumor, etc.	Vendetti et al. ()
		Elimusertib (BAY1895344)	IC50 = 7 nM	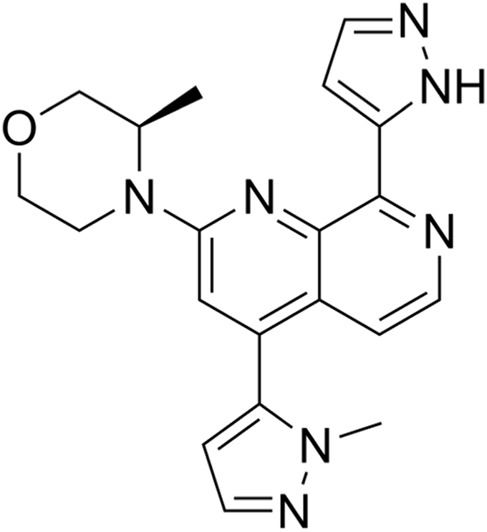	Advanced solid tumors, lymphoma, etc.	Ulrich et al. (2017)
		Gartisertib (VX-803)	IC50 = 8 nM	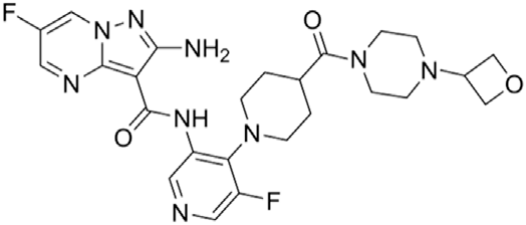	Advanced solid tumors	Zenke et al. (2019)
CHK1 inhibitors	S, G2, and M phases	SCH900776 (MK-8776)	IC50 = 3 nM	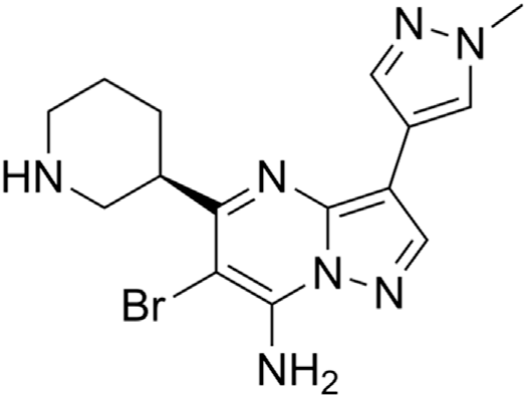	Acute leukemia, advanced solid tumors, etc.	Guzi et al. (2011)
		Prexasertib (LY2606368)	IC50 = 8 nM	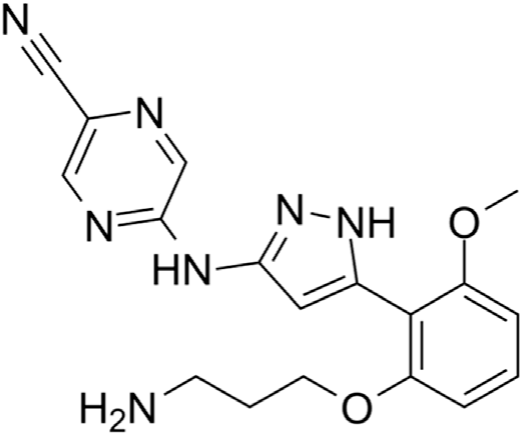	SCLC, platinum-resistant ovarian cancer, some solid tumors, breast cancer, prostate cancer, NSCSC, etc.	King et al. (2015)
		CCT245737 (SRA737)	IC50 = 1.3 nM	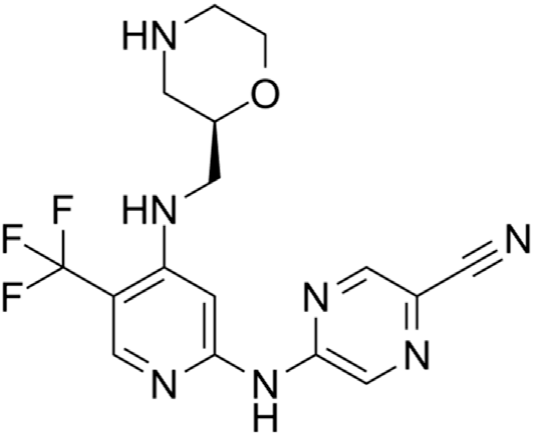	SCLC	Osborne et al. (2016)
Wee1 inhibitors	G2 phase	Adavosertib (AZD1775)	IC50 = 5.2 nM	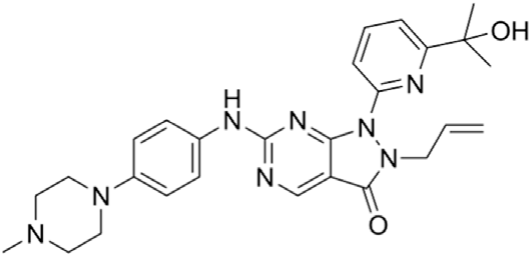	Ovarian cancer, SCLC, solid tumors, metastatic colorectal cancer, NSCLC, TNBC, acute myeloid leukemia, MDS, etc.	Bridges et al. (2011)
CDK1 inhibitors	G2/M phase	Ro-3306	Ki = 20 nM	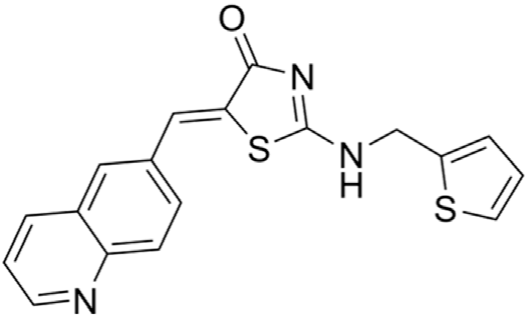	TNBC, ovarian cancer, etc.	Vassilev et al. (2006)
BUB1 kinase inhibitors	M phase	BAY 1816032	IC50 < 7 nM	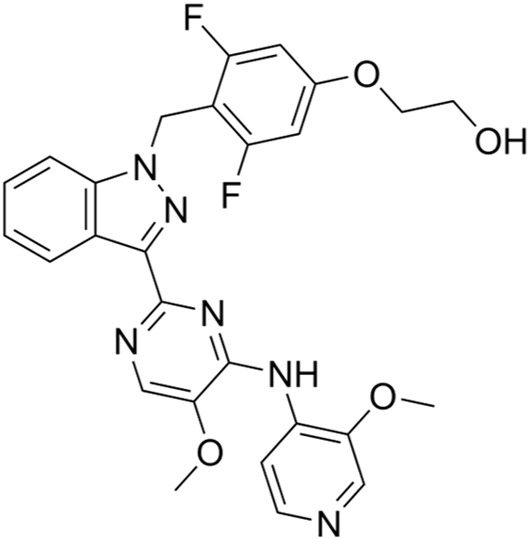	Cervical cancer, TNBC, NSCLC, glioblastoma, prostate cancer, osteosarcoma, etc.	Siemeister et al. (2017)
Inhibitors of tubulin synthesis	M phase	Indibulin	IC50 = 1 50 nM	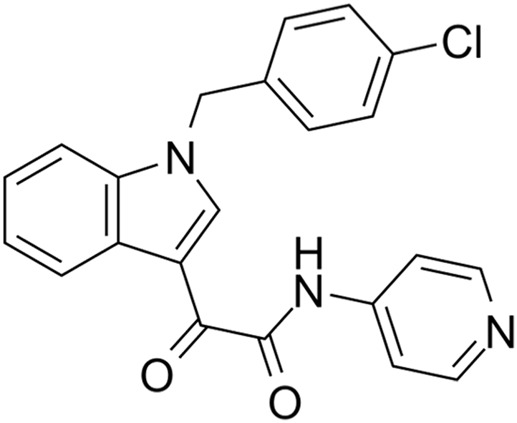	NSCLC, gastric cancer, breast cancer, head and neck cancer, etc.	Kapoor et al. (2018b)
CENP-E inhibitors	M phase	GSK923295	Ki = 3.2 ± 0.2 nM or 1.6 ± 0.1 nM	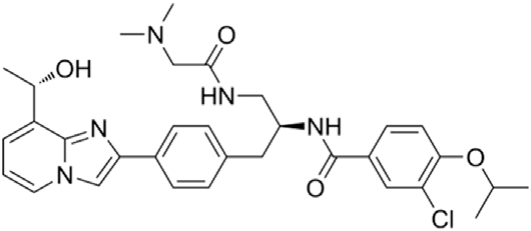	Solid tumors, hematological malignancies, etc.	Wood et al. (2010)

## 2 Differences between normal and malignant cells

In normal cells, the progression and termination of the cell cycle are determined by the cell cycle regulatory system (Khan and Wang, 2022). Once a given cell cycle ends, the commencement of the next cell cycle is dependent upon the needs of the body. Unlike normal cells, malignant tumor cells are relatively autonomous. Owing to the instability of the genome, regulated growth of the malignant tumor cells is disrupted, cell replication is dysregulated by the body, and cell apoptosis is hindered (Hanahan and Weinberg, 2011), which manifests as a cell cycle disorder. Studies have shown that cell cycle disorders in malignant tumors are associated with the cell cycle checkpoints. Malignant tumor cells can overcome the limitations of these checkpoints (Suski et al., 2021) by activating various signaling pathways and altering the expression levels of the intracellular proteins (Li et al., 2024; Zhang et al., 2024; Zhou et al., 2024), eventually leading to imbalances in cell cycle regulation.

## 3 Changes in the cyclin–CDK complexes during different cell cycle phases

### 3.1 G1 phase

The cell cycle ends when the last cell completes mitosis. Then, if the cell needs to undergo another round of mitosis, the new daughter cells will enter the G1 phase and start a new cell cycle. In the G1 phase, the cells synthesize proteins (such as the protein replication complex (pre-RC)), RNA, ribosomes, and other substances in preparation for DNA synthesis in the next phase (Bandura and Calvi, 2002). The cyclinD–CDK4/6 complex plays an important role in this phase; CDK4/6 binds to cyclinD to form a complex, which further activates CDK4/6 to continue the cell cycle. The cyclinD–CDK4/6 complex binds to members of the retinoblastoma (RB) protein family and phosphorylates them. The phosphorylated RB proteins stimulate downstream signaling pathways to release E2F transcription factors and activate the E2F-responsive genes. The E2F-mediated gene expression generates cyclinE, which interacts with CDK2 to form the cyclinE–CDK2 complex that phosphorylates RB, further activating the E2F genes and promoting the progression of the cell division cycle (Wang, 2022; Hamilton and Infante, 2016). The cyclinD–CDK4/6 and cyclinE–CDK2 complexes phosphorylate the RB proteins sequentially to release the restriction of the G1/S checkpoint (Liu et al., 2022); the cells then enter the S phase after passing the G1/S checkpoint.

### 3.2 S phase

In the S phase, the E2F-mediated gene expression stimulates the synthesis of a large number of proteins, and the generated cyclinA and CDK2 form the cyclinA–CDK2 complex (Hume et al., 2020). Moreover, the cyclinE–CDK2 and cyclinA–CDK2 complexes further activate CDK, eventually activating pre-RC and initiating DNA replication. The cells in the S phase complete DNA replication and then enter the G2 phase (Bandura and Calvi, 2002).

### 3.3 G2 phase

During the G2 phase, proteins and other substances are synthesized, and the cells grow in preparation for the next stage of mitosis (Wang, 2022). In the G2 phase, CDK1 interacts with cyclinA to form the cyclinA–CDK1 complex, which plays an irreplaceable role in the cell cycle (Santaguida and Nepveu, 2005). The cyclinA–CDK1 complex activates and stabilizes the cyclinB–CDK1 complex, steadily increasing the activity of the cyclinB–CDK1 complex and advancing the cell cycle to the next stage until mitosis is completed (Wang, 2022). The G2/M phase checkpoint is responsible for detecting DNA damage in the cells; DNA damage that occurs during DNA replication needs to be repaired before the cells enter the next stage to prevent errors in the genetic material (Khan and Wang, 2022).

### 3.4 M phase

**FIGURE 1:**
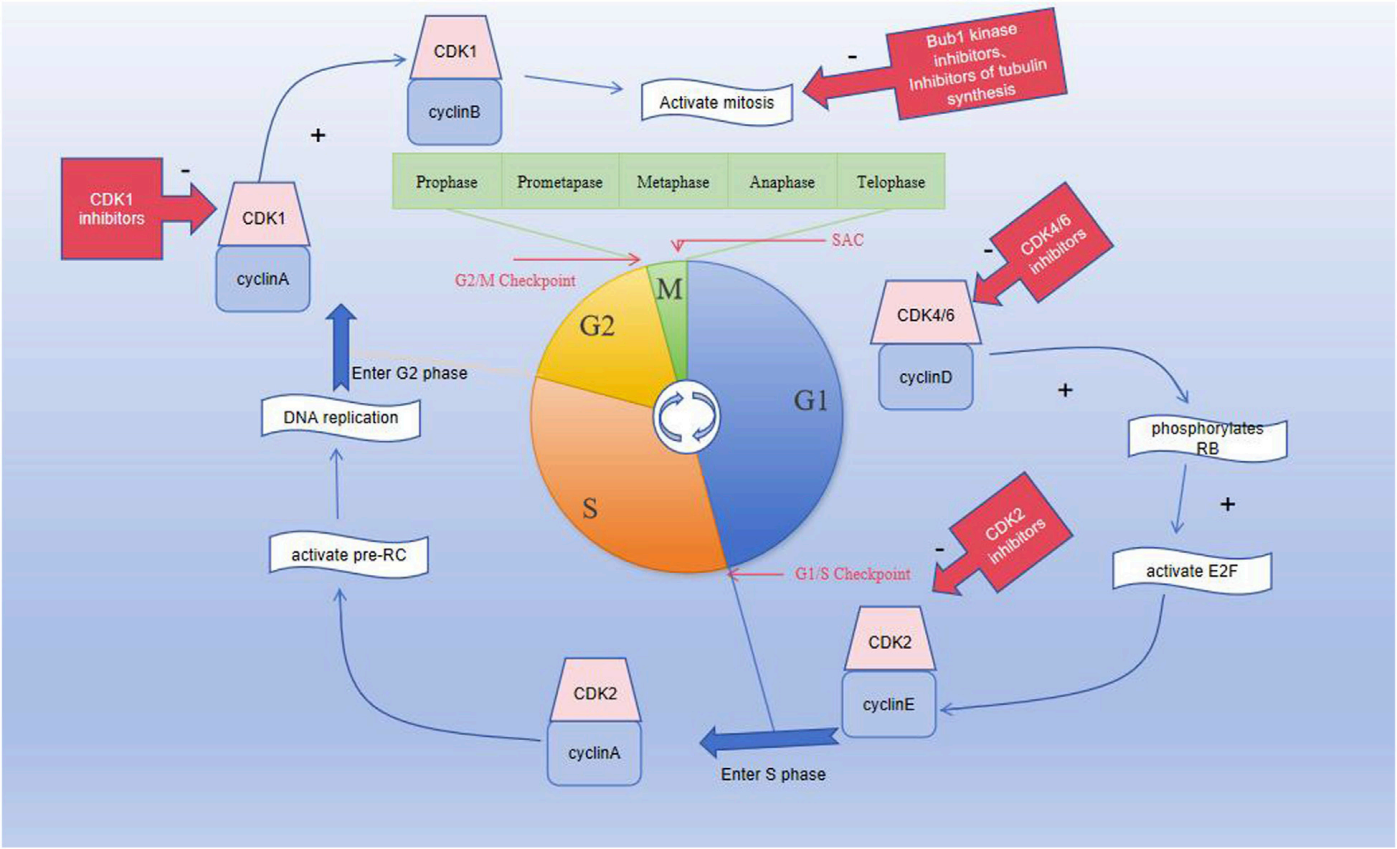
Graph showing the division of the cell cycle into G1, S, G2, and M phases; the M phase is further divided into prophase, prometaphase, metaphase, anaphase, and telophase. Three important checkpoints exist in the cell cycle, namely, the G1/S, G2/M, and spindle assembly checkpoints.

The M phase is divided into mitotic and cytoplasmic divisions; here, mitosis is further divided into prophase, prometaphase, metaphase, anaphase, and telophase. Prophase is characterized by intracellular chromatin/chromosome aggregation, centrosome separation, and nuclear membrane rupture (Figure 1); once the chromosomes in the cell are condensed, the centrosomes separate and move toward the poles of the cell, following which the nuclear membrane ruptures. During the prometaphase, the spindle microtubules attach to the centromere of the chromosomal centromere. During metaphase, the centromere microtubules pull the chromosomes and align them along the equatorial plate to ensure accurate chromosomal separation. During this stage, SAC confirms that the chromosomes are in the proper positions along the equatorial plate, in addition to ensuring that the chromosomes are properly connected to the spindles (Poon, 2016) and that the microtubules are pulling in the correct direction. After passing the SAC, the cell cycle continues and enters the mitotic anaphase. During anaphase, the microtubules pull the chromatids toward each pole. During telophase, the chromosomes uncoil to become chromatin, and the nuclear membrane is formed again. Eventually, the cell membrane contracts inward, dividing the cytoplasm equally between the two cells and forming the two new daughter cells with identical genetic material (Wang, 2022). The cyclinB–CDK1 complex has an irreplaceable role in mitosis, and its activation is believed to trigger mitosis. The cyclinB–CDK1 complex is activated during prophase, degraded in the middle stage, and dephosphorylated in the late stage, until it drops below the threshold and the cell exits mitosis (Gavet and Pines, 2010).

## 4 DNA replication and damage repair during the cell cycle

Although DNA replication has high fidelity, the cells are affected by various damaging factors at all times, making DNA damage inevitable. The DNA-damaging factors can be divided into endogenous and exogenous factors. The endogenous factors include errors in base pairing during DNA replication, instability in the DNA structure, and reactive oxygen species produced by metabolism, among others. The exogenous factors can be roughly divided into biological, chemical, and physical factors. Biological factors mainly include viruses that can reverse transcribe directly to affect the DNA or its metabolites, damaging the integrity of the DNA. The common physical factors include electromagnetic radiation like X-rays, gamma rays, and ultraviolet rays. The chemical factors include free radicals, base analogs, and alkylating agents (Carusillo and Mussolino, 2020). DNA damage activates the DNA damage repair pathway in the body, and the intracellular checkpoint proteins are activated to provide the necessary time for DNA repair, following which the cell cycle continues. If the DNA damage exceeds the limit of repair, the cell will activate the apoptotic pathway and eventually undergo cell death. If the mutated DNA is not repaired and cell replication is not halted, the damaged genetic material will be passed on to the next generation of daughter cells, which will eventually develop into malignant cells. Cancer is believed to result from mutations in cellular DNA through mutations occurring in only a few genes (Basu, 2018).

During the replication of malignant cells, complete DNA replication depends on normal progression of the DNA replication forks. The arresting or deceleration of replication fork progression is called as replication stress, which is an important cause of instability of the genetic material in malignant tumor cells. Damaged DNA not only affects the progression of the DNA replication forks but also causes replication stress (da Costa et al., 2023). However, malignant tumor cells do not die easily from DNA damage because they have special damage repair mechanisms. Malignant tumor cells can repair damaged DNA replication forks through break-induced replication (BIR), thereby ensuring the integrity of the genetic material (Costantino et al., 2014). Understanding the specific DNA repair mechanisms of malignant tumors is thus helpful for identifying the causes of the difficulty with curing malignant tumors and chemotherapeutic resistance, providing new ideas for the development of targeted drugs in the future.

## 5 Cell cycle checkpoints and target drugs

### 5.1 G1/S checkpoint and target drugs

**FIGURE 2:**
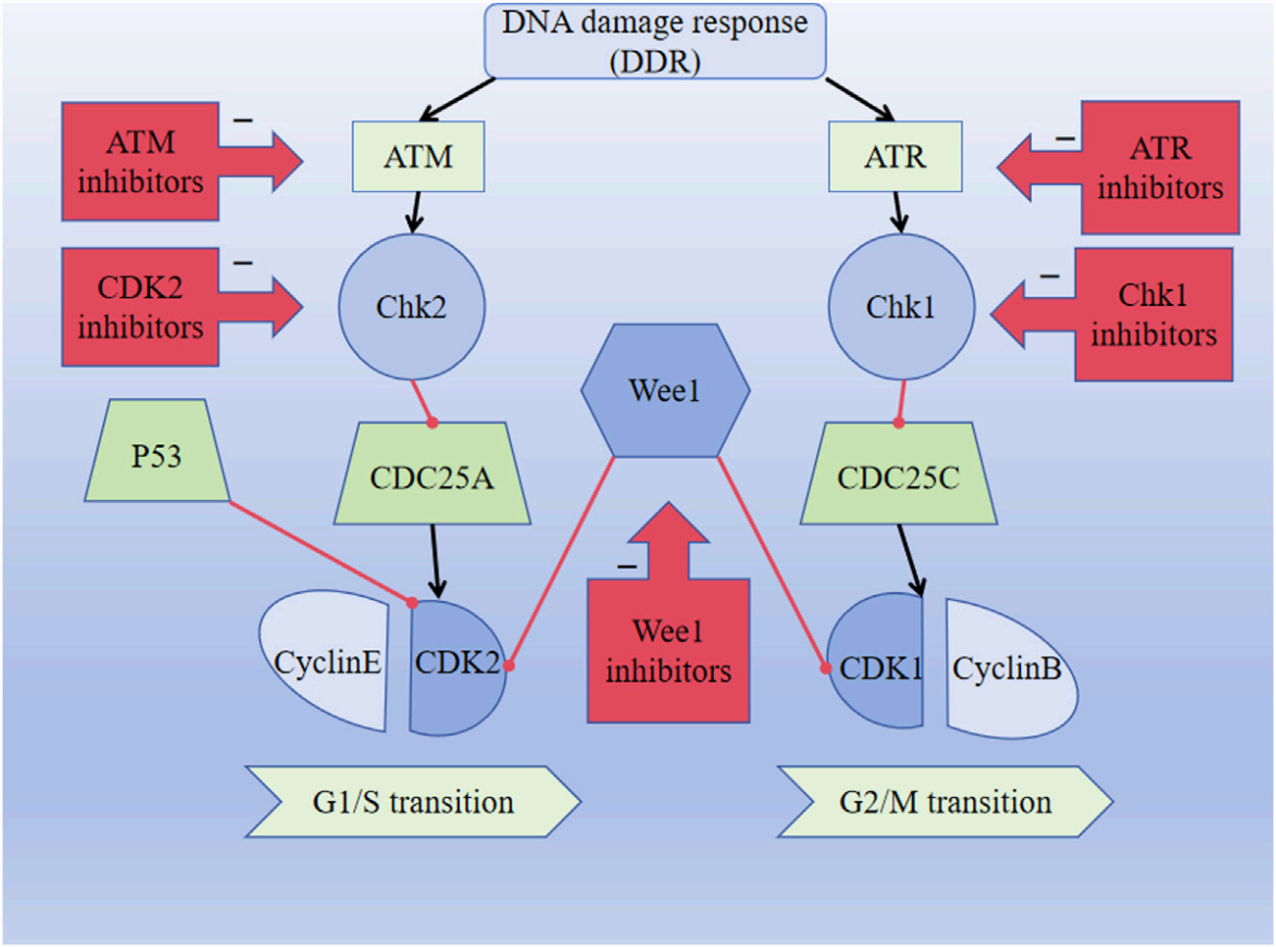
DNA damage response (DDR) pathway activates the ATM-CHK2-P53 signaling pathway and inhibits CDK2 expression, thereby affecting the G1/S transition. DDR pathway activates the ATR-CHK1-Wee1 signaling pathway and inhibits CDK1 expression, thus affecting the G2/M transition. The black lines indicate activation, and the red lines indicate inhibition.

The G1/S checkpoint is an important cell cycle checkpoint that can detect DNA integrity. It prevents DNA damage from being replicated and determines whether the cells can easily enter the S phase. Ataxia telangiectasia mutation (ATM) is a type of DNA damage sensor, and the ATM-Chk2-p53 pathway is activated when DNA damage occurs. Activated ATM regulates the activities of CHK2 and P53, thereby affecting DNA repair and cell cycle progression. ATM phosphorylates CHK2, which then inhibits CDC25A dephosphorylation and ultimately inhibits CDK2, leading to cell cycle disruption (Figure 2). Research shows that ATM is lost in gastrointestinal, respiratory, and lymphatic malignancies, suggesting that the absence of ATM is associated with the development of malignant tumors (Smith et al., 2020). Moreover, studies have shown that the cyclinD–CDK4/6 complex is an important part of the cellular transition from G1 to S phases and that the expressions of the cyclinD–CDK4/6 complex change in various tumors, showing abnormal elevation (Hamilton and Infante, 2016; Chen et al., 2003). Blocking the signaling pathway downstream of cyclinD–CDK4/6 prevents RB protein phosphorylation, thereby preventing cells from entering the S phase and undergoing subsequent tumorigenesis (Hume et al., 2020). In conclusion, the ATM-Chk2-P53 pathway, CDK2, and CDK4/6 may constitute breakthroughs for which we can develop targeted drugs against the proteins; this is expected to improve the prognosis of patients with malignant tumors.

ATM inhibitors: Clinical trials of the ATM inhibitors, including M3541, AZD0156, and AZD1390, are underway. In phase I clinical trials, M3541 adjuvant radiotherapy has been used to treat solid tumors; AZD0156 combined with olaparib, irinotecan, fluorouracil, and folinic acid has been used to treat advanced solid tumors; AZD1390 combined with radiotherapy has been used to treat brain tumors (Smith et al., 2020).

CDK2 inhibitors: Indisulam (E7070) is a type of CDK2 inhibitor that inhibits the activation of cyclinE–CDK2 and the cell cycle, causing G1/S arrest. Ziva Pogacar et al. reported that indisulam combined with the CDK4/6 inhibitor palbociclib could cause senescence or death of lung cancer cells (Pogacar et al., 2022).

CDK4/6 inhibitors: At present, three CDK4/6 inhibitors are in clinical use or trials: abemaciclib (LY2835219), palbociclib (PD-0332991), and ribociclib (LEE011) (Hamilton and Infante, 2016). Palbociclib is currently used to treat perimenopausal or premenopausal patients with breast cancer and is relatively effective against hormone-receptor-positive (HR+) breast cancer cells. In patients with partial mantle cell lymphoma (MCL), palbociclib has been found to be clinically beneficial. Ribociclib can be used to treat tumors with abnormal activities of the cyclinD–CDK complex and downstream pathways; some examples include neurological tumors, fat sarcomas, breast cancer, melanoma, and squamous cell carcinoma of the head and neck, with a certain safety profile. Abemaciclib can be used to treat postmenopausal women with breast cancer, with a clinical benefit rate of up to 72% for patients with HR+/HER2 breast cancers (Gao et al., 2020).

### 5.2 G2/M checkpoint and target agents

The G2/M checkpoint is responsible for monitoring DNA damage during cell cycle progression. The DNA damage response (DDR) is crucial for sustaining the integrity of the genetic material, and activation of the DDR pathway is closely coordinated with cell cycle arrest to prevent transmission of DNA damage to the next generation. Ataxia telangiectasia and Rad3 related (ATR) is a DNA damage sensor associated with phosphorylation and dephosphorylation that inactivates the CDKs, resulting in cessation of the cellular replication process. ATR is activated when the DNA is damaged by external or self-harmful factors. At this point, ATR fully activates the cell cycle checkpoint kinase 1 (CHK1) through serine phosphorylation. The overexpression of Wee1 has been observed in malignant tumor cells, including hepatic cell carcinomas, breast cancers, glioblastoma, respiratory tumors, and gastrointestinal tumors. The upregulation of the ATR-Chk1-Wee1 pathway in tumors may indicate poor prognosis, and the ATR-Chk1-Wee1 pathway is likely to be an attractive focus for the treatment of malignancies (Smith et al., 2020). Moreover, CHK1 inhibits the dephosphorylation functions of the CDC25 family of phosphatases and eventually inhibits CDK1, preventing cell entry into mitosis and thereby blocking the activity of the cyclinB–CDK1 complex (Figure 2) (Smith et al., 2010). Dysregulation of CDK1 expression is associated with the development of various malignant tumors and affects the survival probabilities of patients with different tumor types. Drugs targeting the ATR-Chk1-Wee1 pathway and CDK1 may thus be key strategies for the treatment of malignant tumors (Wang et al., 2023).

ATR inhibitors: Clinical trials of ATR inhibitors, including M6620 (VX-970), AZD6738, BAY1895344, and M4344 (VX-803), are underway. In phase I clinical trials, M6620 combined with radiotherapy was used to treat non-small cell lung carcinoma (NSCLC), small cell lung carcinoma (SCLC), and neural endocrine cancers that metastasized intracranially. M6620 combined with gemcitabine, cisplatin, carboplatin, paclitaxel, irinotecan, and other drugs have been used to treat late-stage solid tumors. M6620 combined with radiation and chemotherapy (cisplatin + capecitabine) has been used to treat esophageal cancer. AZD6738 monotherapy has been used for head and neck squamous cell carcinoma, chronic lymphocytic leukemia (CLL), prolymphocytic leukemia (PLL), B-cell lymphomas, chronic myeloid leukemia (CML), or myelodysplastic syndrome (MDS); AZD6738 combined with acalabrutinib has been prescribed for the treatment of non-Hodgkin lymphoma (NHL); AZD6738 combined with gemcitabine has been used in the treatment of late-stage solid tumors; AZD6738 with paclitaxel has been used to treat metastatic tumors for which standard chemotherapy has failed. BAY1895344 single therapy has been utilized for the treatment of late-stage solid tumors and lymphomas. M4344 (VX-803) is available as a standalone agent or is combination with cisplatin, carboplatin, and gemcitabine as a therapy for late-stage solid tumors. In phase I/II clinical trials, AZD6738 combined with carboplatin, olaparib, or durvalumab was used to treat NSCLC, breast cancer, squamous cell carcinoma of the head and neck, and gastrointestinal malignancies; AZD6738 was combined with acalcitoninib to treat refractory CLL. In phase II trials, M6620 combined with avelumab and carboplatin have been shown to treat primary peritoneal cancer and some malignant tumors of the female reproductive system, such as fallopian tube cancer and ovarian cancer; M6620 combined with cisplatin and gemcitabine has been used to treat metastatic urothelial carcinoma; M6620 combined with carboplatin/docetaxel has been used to treat prostate cancer; M6620 combined with gemcitabine has been used to treat recurrent ovarian cancer, primary peritoneal cancer, and fallopian tube cancer; M6620 combined with Irinotecan has been used to treat gastric or gastroesophageal junction cancers and metastatic gastric cancer. AZD6738 alone has been used to treat triple-negative mammary cancer (TNBC); AZD6738 combined with olaparib has been used in the treatment of nephrocellular carcinoma, urinary tract epithelial carcinoma, pancreatic carcinoma, recurrent ovarian carcinoma, SCLC, and carcinoma of the prostate, among others; AZD6738 and durvalumab combination has been used in the treatment of NSCLC, gastric cancer, and melanoma.

CHK1 inhibitors: At present, CHK1 inhibitors like MK-8776 (SCH900776) and LY2606368 (prexasertib) are used in clinical research. In phase I clinical trials, MK-8776 alone or in combination with cytarabine was used to treat acute leukemia; MK-8776 in combination with hydroxyurea was used to treat late-stage solid tumors. LY2606368 (prexasertib) alone or in combination with gemcitabine/pemetrexed or ralimetanib/olaparib/PD-L1 inhibitor was used to treat solid tumors; LY2606368 combined with cisplatin, cisplatin, cetuximab, or radiotherapy was used to treat head and neck cancers; LY2606368 and cytarabine has been used to treat myeloid leukemia. In phase I/II clinical trials, LY2606368 alone or in combination with gemcitabine was used to treat pancreatic cancer; LY2606368 combined with cisplatin or pemetrexed was used to treat NSCLC. MK-8776 as a single agent or in combination with cytarabine has been used to treat acute myeloid leukemia. In phase II clinical trials, LY2606368 monotherapy was used to treat SCLC, platinum-resistant ovarian cancer, some solid tumors, breast cancer, and prostate cancer; LY2606368 combined with pemetrexed has been used to treat NSCLC (Smith et al., 2020). SRA737 is an FDA-approved CHK1 inhibitor. Sen et al. (2019) reported that SRA737 and gemcitabine (LDG) could be combined to treat SCLC and other cancers (Sen et al., 2019).

Wee1 inhibitors: The Wee1 inhibitor AZD1775 (MK-1775) is currently in clinical trial. In phase I clinical trials, AZD1775 monotherapy has been used to treat ovarian cancer, SCLC, and solid tumors. AZD1775/paclitaxel and carboplatin/orapanitan/gemcitabine/cisplatin combinations have been used to treat solid tumors; AZD1775 combined with irinotecan has been used to treat metastatic colorectal cancer. In phase II clinical trials, AZD1775 monotherapy or in combination with carboplatin/taxol has been used to treat SCLC; AZD1775 and other combinations have been used to treat NSCLC; AZD1775 combined with cisplatin has been used for the treatment of TNBC; the combination of AZD1775 and cytarabine has been used to treat advanced acute myeloid leukemia or MDS (Smith et al., 2020).

CDK1 inhibitors: Ro-3306 and CGP74514A are selective CDK1 inhibitors, and the elimination of CDK1 phosphorylation causes cell cycle arrest at the G2/M checkpoint (Ly et al., 2015). Chen et al. (2023) reported that RO-3306 could significantly reduce the multiplication, mobility, and invasiveness of TNBC while increasing the susceptibility of cancer cells to cisplatin and paclitaxel (Chen et al., 2023). Huang et al. (2023) investigated both an ovarian cancer cell line and a high-grade serous ovarian cancer in a genetically engineered mouse model and reported that CDK1 inhibition plays an antitumor role (Huang et al., 2023). Yang et al. (2021) reported that the combination of CGP74514A (CGP) and a broad-spectrum CDK inhibitor flavopiridol (pull pingdu) can synergistically inhibit acute myeloid leukemia cell proliferation and induce apoptosis (Yang et al., 2021).

### 5.3 SAC and target drugs

The SAC is an important mechanism for safeguarding mitotic fidelity to ensure the accuracy of karyotype numbers in meristematic cells. SAC impairment can cause destabilization of the chromosomes and also tumor development (Hosea et al., 2024). The major SAC components include *BUB1*, *BUBR1*, *MAD2*, *CENP-E*, and *CDC20*, among others. Mutations of the SAC components are associated with tumor progression, suggesting that the SAC components may be potential therapeutic targets for the treatment of malignant tumors (Bolanos-Garcia, 2009). BAY1816032 is a selective BUB1 kinase inhibitor, and Siemeister et al. (2019) reported that BAY1816032 can increase tumor cell sensitivity to paclitaxel, ATR inhibitors, and PARP inhibitors. In addition, the combination of BAY1816032 and paclitaxel has synergistic or additive antiproliferative effects during the treatment of malignancies, including cervical cancers, TNBC, NSCLC, prostate cancer, and intracranial malignant tumor cells (Siemeister et al., 2019). Huang et al. (2021) reported that BAY1816032 can significantly reduce the multiplication, aggressiveness, and migration of osteosarcoma cells and could be a novel therapeutic target for osteosarcoma (Huang et al., 2021). Indibulin is an inhibitor of tubulin synthesis that can activate the spindle assembly checkpoint proteins *MAD2* and *BUBR1* to halt the cell cycle. Kapoor et al. (2018a) observed that indibulin has favorable anticancer activity and less neural toxicity in both preclinical animal models and phase I clinical trials of carcinogenic chemistry; derivatives of indibulin have strong antiproliferative effects on different types of tumor cells, such as head and neck tumors, NSCLC, gastric cancer, and breast cancer (Kapoor et al., 2018a). Li et al. (2019) showed that the *CDC20-MAD2* complex could prevent apoptosis by preventing the early biodegradation of cyclin B1; M2I-1 is an MAD2 inhibitor that interferes with the interactions between *CDC20* and *MAD2*, thereby increasing the susceptibility of cancer cells to antiangiogenic drugs such as paclitaxel (Li et al., 2019). GSK923295 is a specific CENP-E inhibitor that causes chromosomal dislocation and interrupts mitotic progression (Qian et al., 2010). Chung et al. (2012) demonstrated that GSK923295 exhibits *in vitro* antitumor activity against several solid tumor cell lines and hematological malignant cell lines; it also exhibits *in vivo* antitumor activity against numerous solid tumor xenograft models, with very few grade 3 or 4 adverse reactions (Chung et al., 2012).

## 6 Traditional chemotherapeutic drugs

Chemotherapy is an important approach to treating malignant tumors. Based on the different mechanisms of operation, commonly used chemotherapy drugs can be classified into the categories listed below.

### 6.1 Drugs affecting DNA biosynthesis

Methotrexate: Methotrexate mainly affects the S phase of the cell cycle and DNA synthesis, thereby inhibiting the growth and proliferation of malignant tumor cells. Methotrexate is a dihydrofolate reductase inhibitor that prevents the conversion of dihydrofolate to tetrahydrofolate, ultimately reducing the synthesis of deoxythymidine acid and leading to impaired DNA synthesis (Olsen, 1991). Methotrexate is commonly used to treat acute leukemia and choriocarcinoma, and the common adverse reactions are myelosuppression, liver damage, and kidney damage, among others.

Fluorouracil: Fluorouracil mainly acts on the S phase of the cell cycle and can affect DNA synthesis in malignant tumor cells to inhibit the growth of malignant tumors. Fluorouracil is converted to fluorouracil deoxynucleotides in the cells, thereby inhibiting deoxythymidylate synthase and preventing the conversion of deoxyuridine acid to deoxythymidylate to interfere with DNA synthesis. Fluorouracil is mainly used to treat malignant tumors of the digestive system (such as colorectal cancer), breast cancer, and head and neck cancers. The common adverse reactions of this drug are bone marrow suppression and gastrointestinal damage (Longley et al., 2003).

Cytarabine: Cytarabine mainly targets the S phase of the cell cycle, affecting DNA synthesis and interfering with DNA replication to kill the malignant tumor cells. Cytarabine is a DNA polymerase inhibitor; after it enters the body, it produces metabolites such as cytarabine triphosphate, resulting in abnormal functions of DNA polymerase and interfering with DNA synthesis and replication to inhibit the proliferation of malignant tumor cells (Faruqi and Tadi, 2023). Cytarabine is commonly used to treat acute leukemia, and its common adverse reactions are myelosuppression and gastrointestinal reactions (Baker et al., 1991).

### 6.2 Drugs affecting DNA structure and functions

Cyclophosphamide: Cyclophosphamide is a cell cycle non-specific drug that acts by disrupting the structure of the cellular DNA. After cyclophosphamide enters the body, it produces phosphoramide nitrogen mustard, which can undergo alkylation reactions with the DNA in cells, cause DNA breakage, and destroy the normal structure and functions of DNA. Cyclophosphamide is commonly used to treat multiple myeloma, leukemia, and solid tumors. The common adverse reactions of this drug are bone marrow suppression, nausea, and vomiting (Emadi et al., 2009).

Platinum-based drugs: Cisplatin is a cell cycle non-specific drug. After entering the body, cisplatin binds to the double strand of the DNA and forms intrastrand/interstrand crosslinks, which can prevent DNA replication or break the DNA strand. Cisplatin is commonly used to treat testicular cancer and ovarian cancer. The common adverse reactions of these drugs include gastrointestinal reactions, bone marrow suppression, and otonephrotoxicity (Dasari and Tchounwou, 2014).

### 6.3 Drugs affecting RNA and protein syntheses

Adriamycin: Adriamycin acts on cells in all phases of the cell cycle, but cells in the S phase are more sensitive to it. Adriamycin is an anthracycline antibiotic that can bind tightly to DNA, affecting not only DNA replication but also RNA transcription and synthesis. Adriamycin is commonly used to treat acute leukemia, breast cancer, and ovarian cancer. Its common adverse reactions are cardiotoxicity, bone marrow suppression, and gastrointestinal reactions (Tsukagoshi, 1988).

Vincristine: Vincristine mainly targets the M phase of the cell cycle and affects cell cycle progression by altering spindle filament formation. When vincristine binds to tubulin, it inhibits microtubule polymerization, blocks the generation of spindle filaments, and eventually leads to cell cycle arrest. Vincristine is commonly used to treat acute leukemia and lymphoma. Its common adverse reactions are neurotoxicity, bone marrow suppression, and gastrointestinal reactions.

Taxanes: Paclitaxel mainly acts on the M phase of the cell cycle and can affect the normal functions of the spindles. When paclitaxel enters the cell, it promotes tubulin polymerization and inhibits its depolymerization, thus causing a loss of function of the spindle and ultimately blocking cell mitosis to inhibit tumor cell growth. Paclitaxel is commonly used to treat ovarian and breast cancers. Its common adverse reactions are myelosuppression, neurotoxicity, and anaphylaxis (Alqahtani et al., 2019).

### 6.4 Other drugs

Minichromosome maintenance (MCM) complex inhibitors have also been developed. The MCM complex is closely linked to DNA duplication, and its dysfunction can lead to the development of malignant tumors. The inhibition of MCM or its downstream signaling pathway is expected to be a therapeutic target for treating malignant tumors (Li et al., 2021; Cao et al., 2020; Tang et al., 2023; Zeng et al., 2021).

## 7 Cell-cycle-targeting drugs reduce chemotherapeutic resistance

With the development of medicine and pharmacology, various chemotherapeutic drugs have been developed and applied clinically, but drug resistance or even multidrug resistance (MDR) can occur in patients during chemotherapy, whose mechanisms are not fully clear. At present, the possible resistance mechanisms listed below are considered.

### 7.1 Mutations in cell cycle regulatory genes

Mutations in the cell cycle regulatory genes may lead to cell cycle disorders, which potentially affect the efficacies of chemotherapeutic drugs and lead to drug resistance. For example, P53 is a protein that regulates the cell cycle and plays an important role in various malignant tumors. Frequent P53 mutations can eliminate the inhibitory effects on tumor cells and increase the DNA repair functions of malignant tumor cells such that the killing effects of chemotherapeutic drugs on DNA are reduced, leading to chemotherapeutic drug resistance (Alvarado-Ortiz et al., 2021).

### 7.2 Abnormal expressions of cyclins or altered functions of cell cycle checkpoints

The efficacy of a chemotherapeutic drug is closely related to the cell cycle of the malignant tumor cells, and interfering with the cell cycle affects the efficacy of the drug. The progression of the cell cycle is related to the expression levels of cyclins and functions of the cell cycle checkpoints. Malignant tumor cells can interfere with cellular processes by regulating the expressions of cyclins or altering the functions of the cell cycle checkpoints, thereby affecting the efficacies of the chemotherapeutic drugs and leading to resistance. For example, cyclinD expression affects the sensitivity of multiple myeloma cells to chemotherapeutic agents (Bustany et al., 2016). The overexpression of cyclinA is significantly associated with resistance to first-line platinum-based chemotherapy in ovarian cancer cells (Cybulski et al., 2015). The activities of cell cycle checkpoint kinases and functions of these checkpoints are altered in lung cancer cells, thereby interfering with cell cycle progression and leading to chemotherapeutic resistance (Ke et al., 2021).

### 7.3 Activation of antiapoptotic mechanisms

In the occurrence and development of malignant tumors, loss of control of the apoptotic signals and even activation of the antiapoptotic mechanisms can occur in tumor cells, which lead to failure of chemotherapy drugs that induce apoptosis (Mohammad et al., 2015). For example, mutations in the CHEK2 gene activate the P53 apoptotic pathway and induce apoptosis in TNMC cells, leading to chemotherapeutic resistance (Luo et al., 2018).

Cell-cycle-targeting drugs have advantages over traditional chemotherapeutic drugs. In particular, targeted drugs can act on target organs with high specificity and have fewer toxic side effects, which can improve the survival rates of patients (Lee et al., 2018). In contrast to traditional chemotherapy drugs, cell-cycle-targeting drugs have certain advantages in overcoming MDR. On the one hand, given the precise actions of cell-cycle-targeting drugs, the genes of the tumor cells do not readily mutate, thereby reducing the occurrence of drug resistance. On the other hand, cell-cycle-targeting drugs act on cell cycle checkpoints and related pathways, do not directly damage the DNA of the malignant tumor cells, reduce the repair of DNA damage by tumor cells, and reduce the occurrence of chemotherapeutic resistance (Wu et al., 2014). Studies have shown that combination therapy not only reduces the toxic side effects of chemotherapeutic drugs on normal cells but also minimizes drug resistance (Mokhtari et al., 2017; Yue et al., 2021). Moreover, eradicating malignant tumor cells as much as possible and shrinking the tumor can reduce the occurrence of drug resistance (Chatterjee and Bivona, 2019). For example, in the treatment of SCLC, the combination of Wee1 inhibitors can ensure efficacy while reducing the side effects of chemotherapy (Meijer et al., 2022). In NSCLC, the use of a Wee1 inhibitor can increase the sensitivity of the tumor cells to sorafenib (Caiola et al., 2018). Ma et al. (2017) reported that the combination of the CDK4/6 inhibitor palbociclib and anastrozole can inhibit the proliferation of tumor cells and reduce drug resistance in patients with ER+/HER2- breast cancers (Ma et al., 2017).

## 8 Conclusions and perspectives

In this review, we summarize the pathological changes to the cell cycle in malignant tumors and the mechanisms of cell-cycle-targeting drugs. The action mechanisms of malignant tumors are very complex and are have not been fully elucidated thus far; these mechanisms are usually characterized by cell proliferation and are not subject to regulation or cell cycle disorders. Studies have shown that cell cycle progression, cell-cycle-related protein (cyclin, CDK, and CDK inhibitor) expressions, and activation of relevant proteins indicate that malignant tumors are potential therapeutic targets. In recent years, major advancements have been achieved in research on cell-cycle-targeting drugs, and some drugs such as the CDK4/6 inhibitors have been licensed for the clinical treatment of malignant tumors. Moreover, the combination of cell-cycle-targeting drugs and traditional chemotherapeutic drugs can significantly increase the therapeutic effects. However, methods to ensure the efficacy and safety of the drugs and resistance to subsequent treatment are still major problems that must be solved.

Therefore, future research efforts need to be focused on elucidating the pathogenesis of malignant tumors and developing cell-cycle-targeting drugs to formulate novel treatment options with increased scientific and clinical value while providing new hope for the treatment of malignant tumors in the future.
